# Study of the Rolling Effect on MoS_2_–Carbon Fiber Density and Its Consequences for the Functionality of Li-Ion Batteries

**DOI:** 10.3390/ma17122825

**Published:** 2024-06-10

**Authors:** Tai-Yu Wu, Xiao-Ru Li, Bo-Chun Chen, Li-Wen Wang, Jia-Hao Wang, Sheng-Yuan Chu, Chia-Chin Chang

**Affiliations:** 1 Department of Electrical Engineering, National Cheng Kung University, Tainan 70101, Taiwan; asdkickyourass5@gmail.com (T.-Y.W.); ilove61138@gmail.com (B.-C.C.); georgeye113@gmail.com (J.-H.W.); 2 College of Environmental Sciences and Ecology, National University of Tainan, Tainan 70101, Taiwan; a0955503325@gmail.com (X.-R.L.); jk220052@gmail.com (L.-W.W.)

**Keywords:** molybdenum disulfide, lithium-ion batteries, anode material, material density

## Abstract

In this study, an electrode slurry composed of molybdenum disulfide (MoS_2_) and vapor-grown carbon fiber (VGCF) prepared through a solid-phase synthesis method was blade-coated onto copper foil to form a thick film as the anode for lithium-ion batteries. In previously reported work, MoS_2_-based lithium-ion batteries have experienced gradual deformation, fracture, and pulverization of electrode materials during the charge and discharge cycling process. This leads to an unstable electrode structure and rapid decline in battery capacity. Furthermore, MoS_2_ nanosheets tend to aggregate over charge and discharge cycles, which diminishes the surface activity of the material and results in poor electrochemical performance. In this study, we altered the density of the MoS_2_–carbon fiber/Cu foil anode electrode by rolling. Three different densities of electrode sheets were obtained through varying rolling repetitions. Our study shows the best electrochemical performance was achieved at a material density of 2.2 g/cm^3^, maintaining a capacity of 427 mAh/g even after 80 cycles.

## 1. Introduction

With the maturity of the Internet of Things (IoT) and the rapid advancement of technology, the electronics industry is experiencing rapid development. Among the electronic devices developed, the application of two-dimensional materials, sensors, and batteries is pervasive. The structure of a lithium-ion battery can be divided into three principal components: cathode, electrolyte, and anode. The anode is the negative electrode of the battery and is responsible for oxidation reactions during discharge. The anode material employed in commercial lithium-ion batteries is carbon-based. Nevertheless, the use of carbon materials presents challenges, such as the formation of dendritic structures on the surface of the lithium metal, which has the potential to affect the cycle life and raises safety concerns [1]. Carbon materials are commonly employed in lithium-ion batteries, including graphite [2], graphene [3], carbon black [4], carbon fibers [5], and carbon nanotubes [6]. Following modifications by the industry and other research teams, the capacitance of carbon-based materials has approached the theoretical limit, leaving little room for further improvement. As a result, research groups are now exploring new types of anode materials with high capacitance and enhanced safety. Novel anode materials, such as silicon [7], transition-metal oxides (TMOs) [8], and transition-metal dichalcogenides (TMDs) [9], have significantly higher theoretical capacities than carbon materials. However, they are confronted with challenges such as high expansion rates and poor cycle life, which need to be overcome [10].

MoS_2_ has emerged as one of the most commonly used transition metal dichalcogenides (TMDs) for anode materials in recent years. MoS_2_ possesses high theoretical capacity (670 mAh/g; graphite only has 372 mAh/g) [11], is cost-effective, and has low toxicity, which has led to it becoming a focus of research to address its drawbacks and enhance its electrochemical performance. In 2012, Zhang et al. [12] synthesized carbon-coated MoS_2_ nanorods using a combination of hydrothermal and chemical vapor deposition (CVD) methods. Firstly, ammonium heptamolybdate tetrahydrate (AHM) and a nitric acid solution were subjected to a hydrothermal reaction to form MoO_3_ nanorods. Then, the MoO_3_ nanorods were heated under a mixed gas of H_2_S and H_2_ (95:5), resulting in the formation of MoS_2_ nanorods. Subsequently, a second chemical vapor deposition was conducted in the presence of N_2_ and C_2_H_2_, leading to the formation of carbon-coated MoS_2_ nanorods. The carbon coating effectively controlled the expansion of MoS_2_ and increased its conductivity. Following 80 cycles of cycling tests, the carbon-coated MoS_2_ nanorods demonstrated a high capacity of up to 621 mA h/g. This research highlights the synthesis of carbon-coated MoS_2_ nanorods as a means to enhance the electrochemical performance of MoS_2_, addressing its limitations and improving its stability and capacity retention during cycling tests. In 2017, Pan et al. [13] synthesized MoS_2_@SnO_2_-SnS/C nanosheet composite material by employing a freeze-drying technique and hydrothermal reaction. The synthesis involved the use of citric acid, L-cysteine, tin chloride, and sodium molybdate to incorporate SnO_2_ between the easily stacked layers of MoS_2_. This prevented stacking during the charging and discharging process, thereby preventing a decrease in the capacity. Following 100 cycles of a current density of 200 mA/g, the composite material exhibited a capacity of 852 mAh/g. The majority of MoS_2_-C electrodes are typically prepared using chemical vapor deposition (CVD) [14,15,16] or hydrothermal methods [17,18,19]. The CVD process is more expensive and relatively hazardous, while the hydrothermal method is time-consuming, and both processes generate toxic gas byproducts that contribute to environmental pollution. Consequently, we employed a solid-phase synthesis conducted at room temperature. This method is low-cost, safe, and rapid, and it does not yield harmful gas byproducts during the process.

In addition, it is important to consider the capacity of the electrode materials, but it is also crucial to assess their stability for cycling, which is contingent upon the density and morphology of the electrode materials. The rolling step is a less frequently discussed aspect of the electrode fabrication process. However, rolling will increase the electrode material density and change the morphology, which will play an important role in the performance of the battery, which is the motivation of the present work. In this study, how the density of electrodes is affected by different times of the rolling process and the corresponding influence on the performance of battery is discussed in detail.

With the gradual advancement of technology, the demand for high-capacity batteries is steadily increasing. Therefore, the development of materials with higher theoretical capacitance than that of carbon-based materials is important. In this study, we create a lithium-ion battery with high capacity, low degradation rate, and a high cycle life based on MoS_2_. We incorporated commonly used carbon fiber, because the structure of carbon fiber is like tree branches and can enhance structural stability. We also added conductive carbon powder to enhance the conductivity. We further modified the overall material density through a rolling process and investigated the electrochemical and physical properties under different material densities.

## 2. Materials and Method

### 2.1. Electrode Slurry Fabrication by Solid-Phase Synthesis

Weigh 2.255 g of MoS_2_, 0.075 g of conductive carbon black (Super P), 0.22 g of vapor-grown carbon fiber (VGCF), 0.2 g of binder (PVDF), and 5.5 mL of NMP solution (total weight of solids: 2.75 g); details of the raw materials are provided in Table 1. Prepare a planetary ball mill with four zirconia balls in a grinding jar. Proceed with planetary milling according to the rotation speed and time specified in Table 2. After milling, the MoS_2_–carbon fiber slurry is obtained.

### 2.2. Thick Film Deposited by Blade-Coating

Place the copper foil onto the blade-coater and use a paintbrush to flatten the copper foil. Slowly spread the MoS_2_–carbon fiber slurry onto the foil using a medicine spoon and activate the switch to start the coating process. Monitor the changes in the slurry on the copper film during the coating process and add more slurry as needed. Finally, dry the coated foil in an oven for one hour. The obtained product is copper foil with a MoS_2_–carbon fiber thick film.

### 2.3. Electrodes Sheet Fabrication

Cut the copper foil coated with the slurry into small strips using a paper cutter. Place the strips into a rolling machine (RL-3000A, UBIQ, Taoyuan, Taiwan) with a rolling gap of 0.01 mm and a rolling speed of 1 m/min and roll them 0/1/2 times, respectively. Retrieve the copper foil strips and use a punching machine (provided by Hao-Ju Industrial Co., Ltd., Tainan, Taiwan) to cut them into circular discs with a diameter of 1.33 cm. Then, use a feeler gauge to measure the film thickness. After 0/1/2 rounds of rolling, the film thicknesses are 30/14/9 μm, respectively. Finally, weigh the electrodes. We calculate the material density to be 1.02 g/cm^3^, 2.2 g/cm^3^, and 3.43 g/cm^3^, respectively. These discs serve as the negative electrode (anode) of the battery. Place the electrode plates into a vacuum oven (provided by Mingyu Enterprise Co., Ltd., Weifang, China) set at 60 °C and evacuate the chamber for 12 h. Afterward, transfer the plates into a glove box with controlled humidity below 10 ppm. At the end of this step, anode sheets are obtained with different material densities based on the number of rolling times.

### 2.4. Coin Cell Assembly

To assemble the half-cell using a CR2032 coin cell component (provided by Hao-Ju Industrial Co., Ltd.), follow these detailed steps:

1. Inside the glove box, use a lithium metal disc as the positive electrode.
2. Sequentially stack the following components: cell bottom cover, lithium metal (99.9%, FMC), and pre-wetted separator membrane (Celgard 2500) with electrolyte (1 M LiPF6 in EC/DEC/EMC (3:2:5) solution) applied.
3. Use a dropper to add a few drops of electrolyte into the assembly.
4. Proceed by placing the MoS_2_–carbon fiber negative electrode plate, spacer, and cell top cover.
5. Seal and lock the assembly using a capping machine (provided by Hao-Ju Industrial Co., Ltd.).
6. Once sealed, transfer the assembly out of the glove box for storage and subsequent charge–discharge testing.

**Figure 1:**
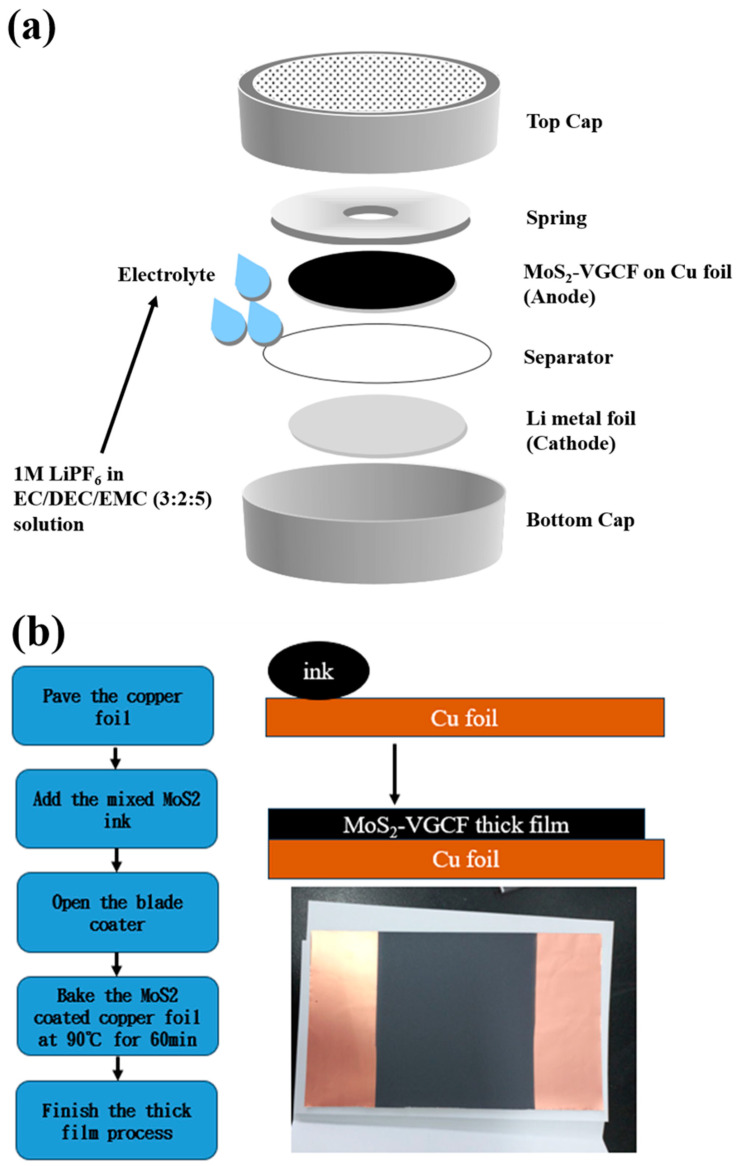
(**a**) Schematic diagram of coin cell assembly. (**b**) Schematic diagram of fabricating MoS_2_–carbon fiber thick films.

Refer to Figure 1a for the assembly process diagram. After the steps are completed, MoS_2_–carbon-fiber-based Li-ion batteries with different material density can be obtained. Figure 1b shows the schematic diagram of fabricating MoS_2_–carbon fiber thick films.

### 2.5. Characterization Techniques

A Raman spectrometer (HORIBA iHR550 System, Tempe, AZ, USA) is used to determine the number of layers in the two-dimensional materials. SEM (Hitachi SU8000, Tokyo, Japan) is used to confirm the morphology. EDS analysis determines the proportions of the elemental compositions. XPS (PHI VersaProbe 4, ULVAC-PHI, Kanagawa Prefecture, Japan) is used to analyze the film composition and defect content. XRD (Bruker D2 PHASER, Billerica, MA, USA) is used to analyze the crystal phase. An electrochemical workstation (Acutech Systems BAT-750B, Gaithersburg, MD, USA) is used to measure the electrochemical properties.

## 3. Results and Discussion

### 3.1. SEM Images of MoS_2_–Carbon Fiber Anodes with Different Material Density

**Figure 2:**
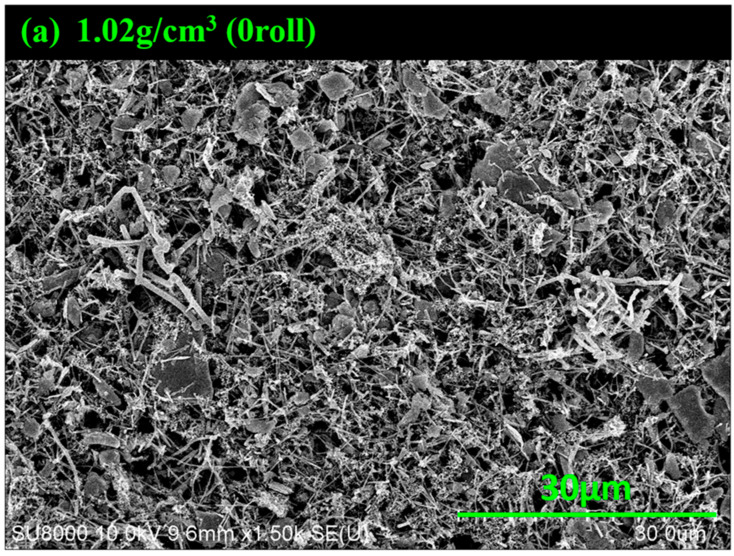

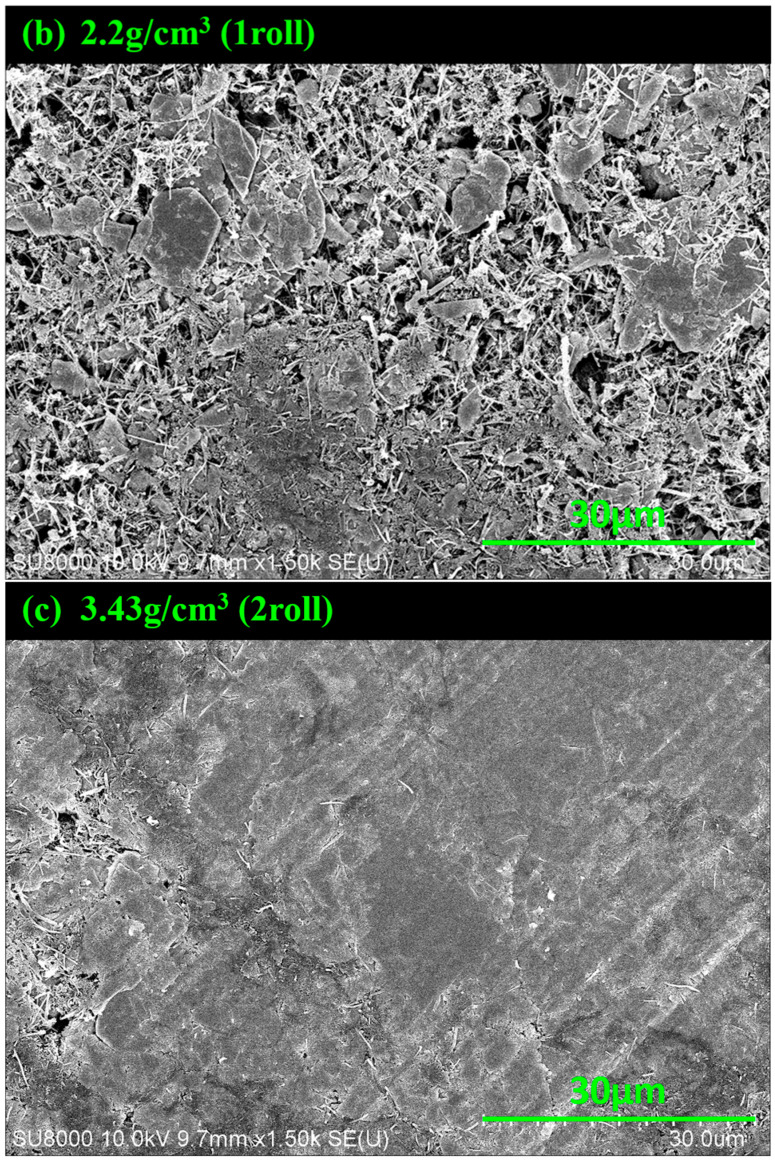
SEM images of MoS_2_–carbon fiber anodes with different material density before charge/discharge cycle. (**a**) 1.02 g/cm^3^; (**b**) 2.2 g/cm^3^; (**c**) 3.43 g/cm^3^.

Figure 2a–c show the SEM images of electrodes with different numbers of rolling. It can be observed that the rolling action changes the morphology of the electrode. Without rolling, the carbon fiber appears like branches with MoS_2_ attached to them. We calculate the material density to be 1.02 g/cm^3^. After rolling once, the morphology changes significantly—some parts become flattened while others still resemble branches. The material density is increased to 2.2 g/cm^3^. For the electrode during twice rolling, the morphology of the surface becomes flatter, and the carbon fiber branches almost disappear. The material density reaches 3.43 g/cm^3^. As indicated by Figure 2a–c, it is evident that the rolling process does indeed lead to changes in the surface morphology and material density.

### 3.2. Raman Spectra Results of MoS_2_–Carbon Fiber Anodes with Different Material Density

**Figure 3:**
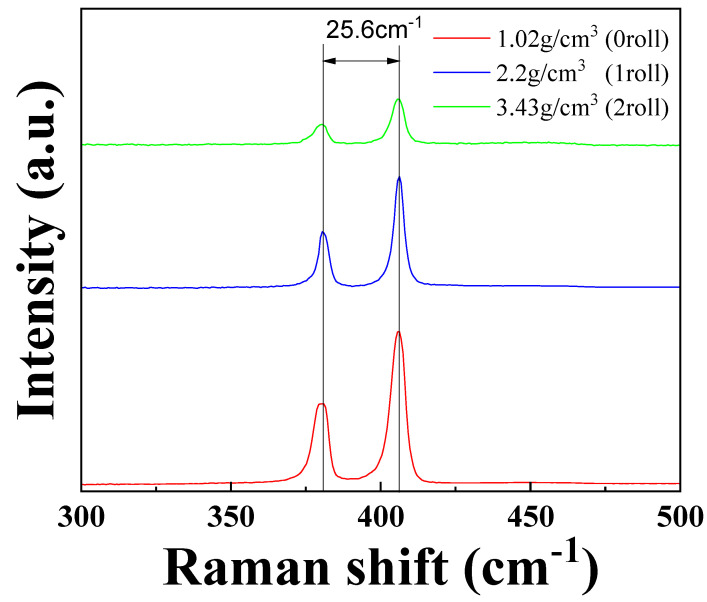
Raman spectra results of MoS_2_–carbon fiber anodes with different material density.

Figure 3 shows the Raman spectroscopy analysis of the electrodes with different material densities. The layer number of MoS_2_ can be estimated from the distance between the E_1g_ and A_2g_ peaks, with the distance between the peaks increasing as the layer number of MoS_2_ increases [20]. The distance between the two peak positions remains at 25.6 cm^−1^, showing no notable variation. It is evident that 25.6 cm^−1^ is already indicative of a thick multilayer or bulk MoS_2_ thickness [21]. The reason for the lack of difference in Raman measurements is that even after multiple rolling cycles, the thickness of the MoS_2_ material, which is only 0.65 nm per layer, has already reached bulk-like thickness. This causes the distance between the two peak positions to remain the same. On the other hand, the intensity of the Raman signals is influenced by the degree of focus during measurement. Since the rolled samples have a flatter surface, achieving a proper focus is more challenging, leading to weaker Raman signal intensity.

### 3.3. X-ray Photoelectron Spectroscopy Results of MoS_2_–Carbon Fiber Anodes with Different Material Density

**Figure 4:**
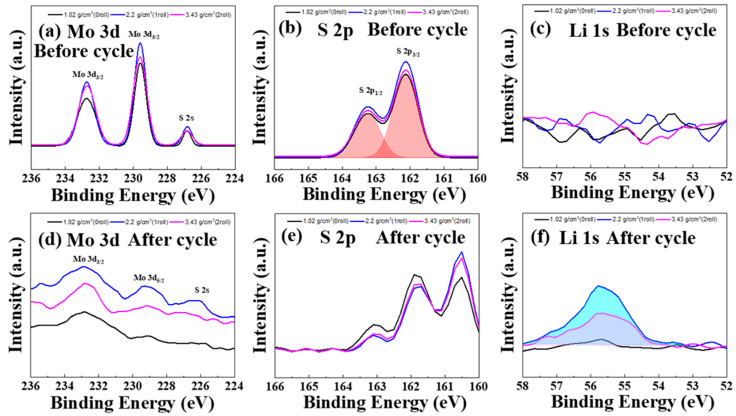

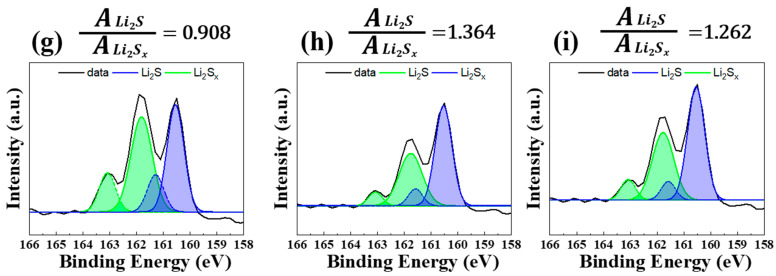
XPS spectra of (**a**) Mo 3d, (**b**) S 2p, and (**c**) Li 1s for MoS_2_–carbon fiber anodes with different material density before cycling; XPS spectra of (**d**) Mo 3d, (**e**) S 2p, and (**f**) Li 1s for MoS_2_–carbon fiber anodes with different material density after cycling; XPS spectra of S 2p fitting curve for MoS_2_–carbon fiber anodes with different material density, (**g**) 1.02 g/cm^3^, (**h**) 2.2 g/cm^3^, and (**i**) 3.43 g/cm^3^.

Figure 4a,b present the XPS analysis results of the Mo 3d and S 2p orbitals of our electrode before cycling. It is observed that the MoS_2_ electrodes predominantly consist of intrinsic MoS_2_ [22]. The thick film fabrication process involves the physical preparation of MoS_2_ powder without any chemical transformations. Therefore, the predominance of intrinsic MoS_2_ is expected. Figure 4c presents the XPS analysis results of the Li 1s orbital before cycling. Because the charge/discharge cycles have not been tested yet, the just-prepared electrodes are devoid of lithium. As a result, the lithium peak is not observable in Figure 4c. Because electrode preparation with varying number of rolling times did not involve chemical reactions, the XPS results did not show significant differences between the different material densities of the electrodes, as seen in Figure 4a–c. Figure 4d–f present the XPS analysis results of the Mo 3d, S 2p, and Li 1s orbitals of our electrode after 50 times charge/discharge cycling. From Figure 4d, it can be observed that the molybdenum peak corresponding to MoS_2_ is nearly absent. In Figure 4e, the sulfur peak associated with MoS_2_ is also nearly absent, and in its place, peaks corresponding to Li_2_S and Li_2_S_x_ appear [23]. Figure 4f reveals that the initially lithium-free electrodes undergo a reaction with lithium ions from the electrolyte after the charge/discharge cycles, resulting in the formation of lithium metal on the electrode surface. This phenomenon is more pronounced in the rolled samples. The chemical reaction equations that occur during charge and discharge are according to the following equations: [24,25,26,27]

(1) $${MoS}_{2}+x{Li}^{+}+xe^{-}↔{Li}_{x}{MoS}_{2}\;\;\;\left(∼1.1V\;\mathrm{vs}.\;Li/{Li}^{+}\right)\;0\leq x\leq 1$$

(2) $${Li}_{x}{MoS}_{2}+(4-x){Li}^{+}+(4-x)e^{-}\to Mo+2{Li}_{2}S\left(∼0.6V\;\mathrm{vs}.\;{Li}^{2}/{Li}^{+}\right)\;0\leq x\leq 4$$

(3) $$S+2{Li}^{+}+2e^{-}↔{Li}_{2}S\left(∼2.2V\;\mathrm{vs}.\;Li/{Li}^{+}\right)$$

(4) $$Mo+{Li}_{2}S\to {MoS}_{2}+{Li}^{+}+e^{-}$$

Figure 4g–i are the XPS results of the electrodes with different densities (Figure 4g: 1.02 g/cm^3^, Figure 4h: 2.2 g/cm^3^, Figure 4i: 3.43 g/cm^3^, respectively) from Figure 4e, after performing peak deconvolution for the peaks of Li_2_S and Li_2_S_x_. By deconvoluting the peaks, Li_2_S corresponds to the peaks at 160.5 eV and 161.7 eV, while Li_2_S_x_ corresponds to the peaks at 161.9 eV and 163.1 eV [23]. Figure 4g–i show the deconvolution results of Figure 4e for the samples under different rolling times. The composition ratio of Li_2_S and Li_2_S_x_ can be determined through the area under the curves, with Li_2_S represented in blue and Li_2_S_x_ in green. Under the conditions of 0/1/2 rolling cycles, the composition ratios of A and B are 0.908/1.364/1.262, respectively. By observing Figure 4g–i, it can be deduced that rolling promotes a shift in material towards Li_2_S rather than transforming into Li_2_S_x_. This is because once transformed into Li_2_S_x_, further reactions become less likely [28]. Therefore, in electrode compositions with a higher proportion of Li_2_S, the resulting batteries exhibit a better rate of retention.

### 3.4. Formation Results of MoS_2_–Carbon Fiber Anodes with Different Material Density

**Figure 5:**
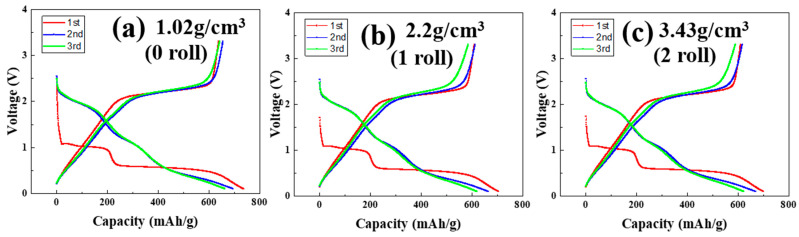
Formation results of MoS_2_–carbon fiber anodes with different material density. (**a**) 1.02 g/cm^3^; (**b**) 2.2 g/cm^3^; (**c**) 3.43 g/cm^3^.

Figure 5a–c and Table 3 represent the formation analysis of the assembled cells using electrodes rolled 0, 1, and 2 times, respectively. The tests were conducted at a constant temperature of 25 °C, with complete charge and discharge cycles at a rate of 0.1 C for three cycles. The cutoff voltage for both discharge and charge are set at 0.1 V and 3.3 V, respectively. During the first three cycles, it is observed that the MoS_2_ material exhibits capacitance values similar to those of conventional carbon-based materials, owing to its high initial theoretical capacitance. The coulombic efficiency, which is the ratio of the charge capacity to the discharge capacity, shows values of approximately 90% for the first three cycles. A comparison among the three devices reveals that the unrolled electrode exhibits a higher capacitance value. This can be attributed to structural factors that make the unrolled electrode more conducive to lithium-ion insertion. However, it may also affect its ability to retain lithium ions. As the number of charge and discharge cycles increases, this drawback will progressively and significantly impact the overall performance of the device.

### 3.5. Cyclic Voltammetry Test Results of MoS_2_–Carbon Fiber Anodes with Different Material Density

**Figure 6:**
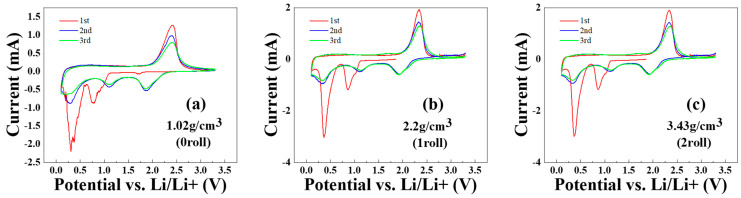
CV test results of MoS_2_–carbon fiber anodes with different material density. (**a**) 1.02 g/cm^3^; (**b**) 2.2 g/cm^3^; (**c**) 3.43 g/cm^3^.

Figure 6a–c show the cyclic voltammetry (CV) measurements of batteries fabricated using electrodes with different rolling numbers. The scan range is from 3.3 V to 0.1 V (at 0.05 mV/s), and each electrode undergoes three cycles. From Figure 6a, it can be observed that the electrode without rolling exhibits unstable contact with the ion-exchange membrane, resulting in erratic initial reduction peak currents. In contrast, Figure 6b,c show improved stability due to the rolling process. Comparing all three figures, a distinct difference is observed between the first cycles and the subsequent cycles.

In the first cycle of CV curves, the first reduction peak at 0.9 V corresponds to the insertion of lithium ions as described in Equation (1) [26,27,28,29]. The reduction peak at 0.4 V corresponds to the formation of Li_2_S as described in Equation (2). The oxidation peak at 2.4 V corresponds to the formation of MoS_2_ as described in Equations (1) and (4).

In the second and third cycle of CV curves, the reduction peak at 1.8 V corresponds to the formation of Li_2_S as described in Equation (3). The reduction peak at 1.2 V corresponds to the formation of Li_x_MoS_2_ as described in Equation (1). The reduction peak at 0.3 V corresponds to the formation of Mo metal as described in Equation (4). The oxidation peak at 2.4 V corresponds to the formation of MoS_2_ as described in Equations (1) and (4).

From the results of the CV test, we can deduce that the oxidation and reduction peaks correspond to the chemical reaction equations in accordance with the XPS results discussed in earlier sections.

### 3.6. Cycle Life Test Results of MoS_2_–Carbon Fiber Anodes with Different Material Density

**Figure 7:**
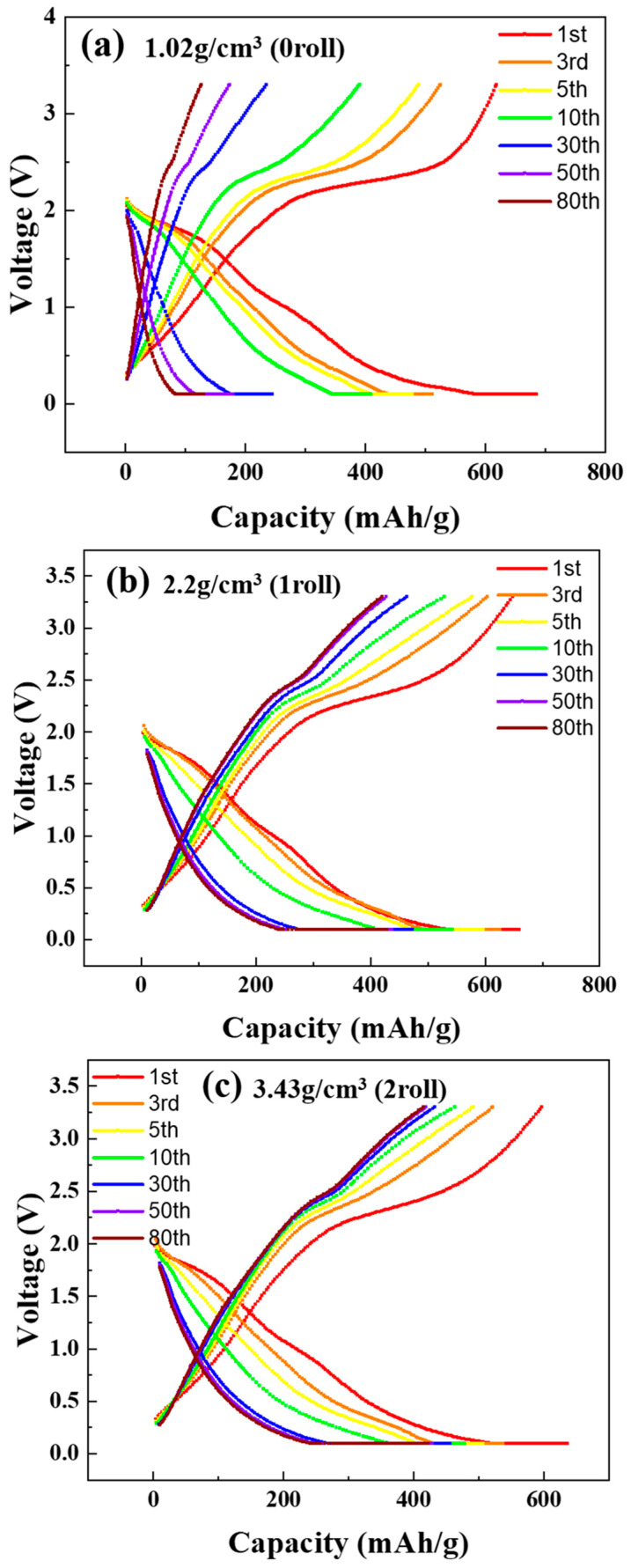
Cycle life test results of MoS_2_–carbon fiber anodes with different material density. (**a**) 1.02 g/cm^3^; (**b**) 2.2 g/cm^3^; (**c**) 3.43 g/cm^3^.

**Figure 8:**
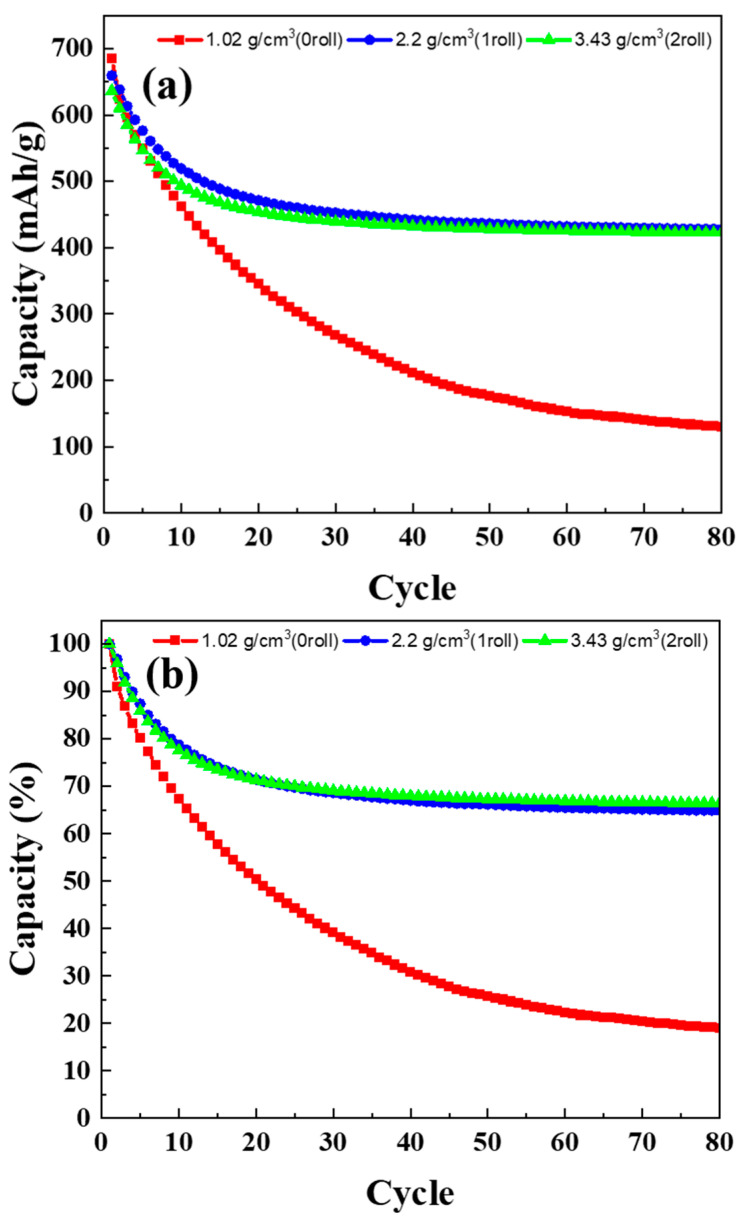
The (**a**) unnormalized and (**b**) normalized capacity retention curves of different anode material density.

Figure 7a–c show the charge–discharge characteristic (under 0.2 C) of batteries fabricated using electrodes with different rolling numbers. The scan range is from 3.3 V to 0.1 V. Figure 8a presents the discharge capacity as a function of cycle number over 80 cycles, while Figure 8b shows the normalized capacity as a percentage. In this section, the batteries were subjected to 80 cycles after formation. The 1st, 3rd, 5th, 10th, 30th, 50th, and 80th cycles were selected for the charge–discharge plots. It can be observed that, in the case of non-rolled electrodes, the initial energy storage capacity is higher at 685 mAh/g. However, this capacity gradually diminishes with increasing charge–discharge cycles, reaching only 130 mAh/g in the 80th cycle. For the electrodes rolled once, the initial capacity is slightly lower at 659 mAh/g, but the capacity retention improves significantly. After 80 cycles, it retains a capacity of 427 mAh/g. In the case of electrodes rolled twice, the initial capacity is 636 mAh/g, and after 80 cycles, it retains a capacity of 422 mAh/g. In terms of percentage retention, the non-rolled, once-rolled, and twice-rolled electrodes retain approximately 18.9%, 64.9%, and 66.4% of their initial capacity, respectively. While the twice-rolled electrodes show better retention as a percentage, the trade-off between capacity and rolling effects must be considered. Based on the trade-off analysis, it is concluded that the electrodes rolled once are more suitable for lithium-ion batteries.

### 3.7. C-Rate Test Results of MoS_2_–Carbon Fiber Anodes with Different Material Density

**Figure 9:**
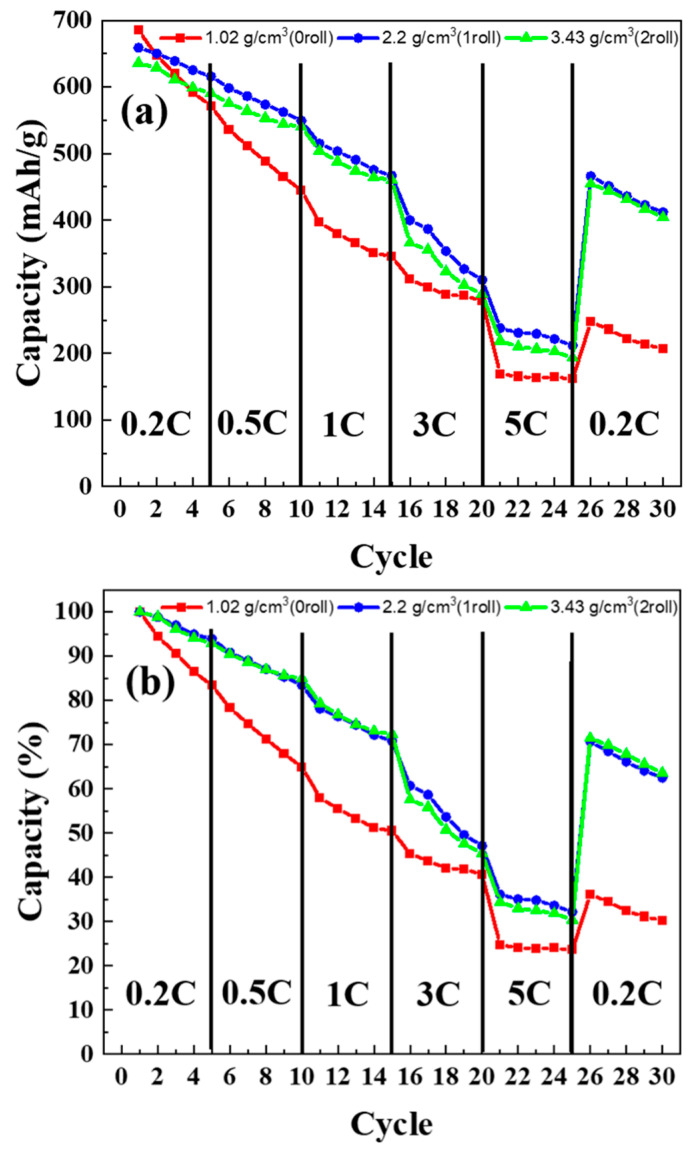
C-rate test results of MoS_2_–carbon fiber anodes with different material density. (**a**) Unnormalized, (**b**) normalized.

Figure 9a presents the discharge capacity under different current density, while Figure 9b shows the normalized capacity as a percentage. Rate capability (C-rate) tests were conducted under discharge conditions of 0.2 C, 0.5 C, 1 C, 3 C, and 5 C, while charging was consistently performed at 0.2 C. The discharge and charge cutoff voltages were set at 0.1 V and 3.3 V, respectively. From Figure 9a,b, it is evident that the electrodes without rolling exhibit unsatisfactory rate capability, while rolling significantly improves this aspect. Comparing the discharge rates with other studies, when compared to carbon-based materials, which show a capacity decline to below 10% at 5 C [29], the MoS_2_ material in this study maintains over 30% capacity (230 mAh/g). This is speculated to be due to the absence of SEI film formation from the conversion mechanism, allowing ions and charges to diffuse rapidly, eliminating the need for a thick SEI film during high-speed charging and discharging.

In the C-rate testing, although electrodes rolled once and twice exhibit similar capacity recovery proportions during the final 0.2 C discharge, the initial higher capacity of the once-rolled electrode results in the highest actual capacity at the end of the test. This aligns with the cycle test results from the earlier section. In summary, based on the findings, it can be inferred that a single rolling cycle is the more optimized condition in this study.

### 3.8. Compared to Other Reported Data

Table 4 presents a comparison between our research and other studies about MoS_2_-based lithium-ion batteries. We compared capacity, number of cycles, and retention rate. Our research demonstrates that our device has higher capacity, and although the retention rate may not be outstanding, the rate capability is exceptional compared to other studies. This indicates that our device can quickly charge/discharge. Therefore, it has significant application potential in fast-charging devices.

## 4. Conclusions

In this study, we investigated an MoS_2_-based anode electrode in lithium-ion batteries. We fabricated different density MoS_2_–carbon fiber thick films by controlling the number of rolling times and assembling a lithium-ion battery. The morphology of the different density MoS_2_–carbon fiber thick films was observed by SEM and the chemical reactions were verified by XPS and CV analysis. Following the long-cycle charge/discharge test, it was observed that rolling the material in question resulted in a reduction in the initial capacity yet led to an enhanced rate of capacity retention. After considering the trade-off, we identified that the optimal strategy for fabricating an MoS_2_-based lithium-ion battery is to roll only once. Finally, we tested our device and found our device can keep a capacity of 427 mAh/g after 80 cycles and can operate under 5 C (3350 mA/g) current density. These characteristics demonstrate the potential for application of our device in the field of high-speed charging.

## Figures and Tables

**Table 1:** List of chemicals.

Material	Chemical Formula	Purity	Manufacturer
Molybdenum disulfide powder	MoS_2_	98%	Sigma-Aldrich, St. Louis, MO, USA
Vapor-grown carbon fiber (VGCF) powder	C	>98%	Showa Denko K.K., Oyama City, Japan
Super P (conductive carbon)	C	>99.5%	TIMCAL, Westlake, OH, USA
N-Methyl-2-pyrrolidone (NMP)	C_5_H_9_NO	99%	Emperor Chemical Co., Ltd., Hangzhou, China
Polyvinylidene difluoride (PVDF)	-(C_2_H_2_F_2_)_n_-	99%	KUREHA, Louisville, KY, USA
Electrolyte: EC/DEC/EMC 3:2:5 (*w:w:w*) + 1 M LiPF_6_	-	-	Taiwan Hopax Chemicals Mfg. Co., Ltd., Kaohsiung, Taiwan
Copper foil	Cu	99.9%	UACJ Co., Tokyo, Japan
Lithium metal	Li	99.9	FMC Co., Philadelphia, PA, USA
Separator (Celegard2300) (polypropylene/polyethylene/polypropylene)	-	-	Celegard LLC, Charlotte, NC, USA

**Table 2:** Ball-milling parameters.

STEPS	Action	Speed (100 rpm)	Time(min)
STEP1	Add PVDF in NMP	3/6/3/7/3	6/6/6/6/6
STEP2	Continue milling	3/6/3/7/3	6/6/6/6/6
STEP3	Continue milling	3/6/3/7/3	6/6/6/6/6
STEP4	Add SP and VGCF	3/6/3/7/3	6/6/6/6/6
STEP5	Add MoS_2_	3/6/3/7/3	6/6/6/6/6
STEP6	Defoaming	1.5	30

**Table 3:** Formation results list of different anode material density.

	0 Roll (1.02 g/cm^3^)	1 Roll (2.2 g/cm^3^)	2 Roll (3.43 g/cm^3^)
1st discharge (mAh/g)	736.6	704.9	700.7
1st charge (mAh/g)	638.7	610.1	610.2
1st coulombic efficiency	86.7%	86.6%	87.1%
2nd discharge (mAh/g)	695.6	664.2	669.3
2nd charge (mAh/g)	655.6	613.3	616.8
2nd coulombic efficiency	94.2%	92.3%	92.2%
3rd discharge (mAh/g)	663.3	620.7	623.7
3rd charge (mAh/g)	642.5	585.7	589.7
3rd coulombic efficiency (mAh/g)	96.8%	94.4%	94.5%
3rd retention	90.0%	88.1%	89.0%

**Table 4:** Our MoS_2_–carbon fiber lithium-ion battery compared to other MoS_2_-based studies.

Material	Capacity (mAh/g)	Cycles (retention%)	Ref.
MoS_2_/graphene	225	200(90%) (@1000 mA/g)	[30]
MoS_2_ nanotubes	260	30(98%) (@50 mA/g)	[31]
MoS_2_/PEO	225	70(66%) (@50 mA/g)	[32]
MoS_2_	85	100(64%) (@50 mA/g)	[33]
MoS_2_–carbon fiber	427	80(64.8%) (@134 mA/g)	This work

## Data Availability

The data that support the findings of this study are available from the corresponding author upon reasonable request.
